# Bowel stimulation before loop ileostomy closure to reduce postoperative ileus: a multicenter, single-blinded, randomized controlled trial

**DOI:** 10.1007/s00464-022-09510-5

**Published:** 2022-08-19

**Authors:** Richard Garfinkle, Marie Demian, Sarah Sabboobeh, Jeongyoon Moon, Michael Hulme-Moir, A. Sender Liberman, Stan Feinberg, Dana M. Hayden, Sami A. Chadi, Sebastian Demyttenaere, Louise Samuel, Nevart Hotakorzian, Laurence Quintin, Nancy Morin, Julio Faria, Gabriela Ghitulescu, Carol-Ann Vasilevsky, Marylise Boutros, Bowel Stimulation Research Collaborative, John Jarvis, Andrew Herd, Andrew Moot, Siraj Rajaratnam, Sherry Nisbet, Patrick Charlebois, Lawrence Lee, Barry Stein, Peter Stotland, Usmaan Hameed, Anuradha R Bhama, Fayez Quereshy, Donna Tataryn

**Affiliations:** 1 Division of Colon and Rectal Surgery, Jewish General Hospital, Montreal, QC Canada; 2 Department of Surgery, North Shore Hospital, Auckland, New Zealand; 3 Department of Surgery, McGill University Health Center, Montreal, Canada; 4 Department of Surgery, North York General Hospital, Toronto, Canada; 5 Division of Colon and Rectal Surgery, Rush University Medical Center, Chicago, USA; 6 Department of Surgery, University Health Network, Toronto, Canada; 7 Department of Surgery, St. Mary’s Hospital, Montreal, Canada; 8 Department of Nursing, Jewish General Hospital, Montreal, Canada; 9 Division of Colon and Rectal Surgery, Jewish General Hospital, McGill University, 3755 Cote Ste Catherine, G-317, Montreal, QC H3T 1E2 Canada

**Keywords:** Bowel stimulation, Loop ileostomy closure, Postoperative ileus, Length of stay

## Abstract

**Introduction:**

The objective of this study was to evaluate the impact of preoperative bowel stimulation on the development of postoperative ileus (POI) after loop ileostomy closure.

**Methods:**

This was a multicenter, randomized controlled trial (NCT025596350) including adult (≥ 18 years old) patients who underwent elective loop ileostomy closure at 7 participating hospitals. Participants were randomly assigned (1:1) using a centralized computer-generated sequence with block randomization to either preoperative bowel stimulation or no stimulation (control group). Bowel stimulation consisted of 10 outpatient sessions within the 3 weeks prior to ileostomy closure and was performed by trained Enterostomal Therapy nurses. The primary outcome was POI, defined as an intolerance to oral food in the absence of clinical or radiological signs of obstruction, on or after postoperative day 3, that either (a) required nasogastric tube insertion; or (b) was associated with two of the following: nausea/vomiting, abdominal distension, or the absence of flatus.

**Results:**

Between January 2017 and November 2020, 101 patients were randomized, and 5 patients never underwent ileostomy closure; thus, 96 patients (47 stimulated vs. 49 control) were analyzed according to a modified intention-to-treat protocol. Baseline characteristics were well balanced in both groups. The incidence of POI was lower among patients randomized to stimulation (6.4% vs. 24.5%, *p* = 0.034; unadjusted RR: 0.26, 95% CI 0.078–0.87). Stimulated patients also had earlier median time to first flatus (2.0 days (1.0–2.0) vs. 2.0 days (2.0–3.0), *p* = 0.025), were more likely to pass flatus on postoperative day 1 (46.8% vs. 22.4%, *p* = 0.022), and had a shorter median postoperative hospital stay (3.0 days (2.0–3.5) vs. 4.0 days (2.0–6.0), *p* = 0.003).

**Conclusions:**

Preoperative bowel stimulation via the efferent limb of the ileostomy reduced POI after elective loop ileostomy closure.

**Supplementary Information:**

The online version contains supplementary material available at 10.1007/s00464-022-09510-5.

Loop ileostomies are commonly employed for temporary protection of high-risk colorectal anastomoses [1]. However, a second operation is required to restore intestinal continuity, and loop ileostomy closure is associated with considerable postoperative morbidity [2]. Of all complications following closure, postoperative ileus (POI) is the most common [3], with large observational studies reporting incidences ranging from 10 to 20% [4–6]. POI is also a leading cause of 30-day readmission following loop ileostomy closure [7–9], particularly in the context of modern Enhanced Recovery Protocols (ERP) with early, or even same-day, discharge.

While transient gut dysmotility can be expected after any gastrointestinal operation, in the case of an ileostomy, the defunctionalized segment of bowel undergoes a series of structural and functional changes that may increase the time it takes for motility to resume after closure. Studies have demonstrated that defunctionalization of the ileum leads to atrophy of its villi and muscular layers, as well as changes in gut hormone production and the absorptive capacity in the distal bowel segments [10–12]. These functional and structural changes may provide a unique target for preoperative intervention in an attempt to improve postoperative return of bowel function. In 2014, Abrisqueta et al. published a single-center randomized controlled trial of 70 patients, evaluating the impact of preoperative bowel stimulation through the efferent limb of the ileostomy on POI after loop ileostomy closure. They demonstrated a decreased rate of POI in the stimulated group (3% vs. 20%, *p* < 0.001) and a 2-day reduction in postoperative length of stay [13]. To date, this was the only randomized trial to assess bowel stimulation before ileostomy closure.

The purpose of this study was to evaluate the impact of preoperative bowel stimulation on the development of POI after loop ileostomy closure.

## Materials and methods

### Study design

This was an international, multicenter, pragmatic, single-blinded, parallel-group superiority trial evaluating the impact of preoperative bowel stimulation versus no intervention (control group) on the development of POI after elective loop ileostomy closure. Patients were recruited from 7 participating hospitals in Canada (5 centers), the United States (1 center), and New Zealand (1 center) (Supplementary Table 1). The Research Ethics Board at the Jewish General Hospital (Montreal, QC, Canada) approved the study and acted as the primary review board; approval was further obtained at each participating hospital prior to recruitment. The trial was pre-registered online (NCT02559635) and the study protocol was previously published [14]. The study was reported in accordance with the CONSORT statement. [15]

### Participants

Adult patients (≥ 18 years old) who had undergone a segmental colectomy or proctectomy with a diverting loop ileostomy and were being consented for elective loop ileostomy closure were offered inclusion into the study, provided they could be expected to attend the outpatient simulation sessions prior to their operation. Patients who underwent a total abdominal colectomy or total proctocolectomy, those with underlying Crohn’s disease or known peritoneal metastases at the time of ileostomy closure, those undergoing a planned midline laparotomy and/or concomitant procedure at the time of ileostomy closure, or those in whom clear and informed consent could not be obtained were not eligible for study inclusion. All patients underwent radiographic (± endoscopic) confirmation of distal anastomotic healing prior to recruitment.

### Randomization and allocation concealment

Parallel-group (1:1) block randomization was performed to ensure a similar number of participants in both groups. Randomization was stratified by the operative approach of the index operation (open vs. minimally invasive), as POI and overall 30-day postoperative morbidity after loop ileostomy closure were demonstrated to be higher after an open index colorectal resection [3, 16]. Randomization was performed using a computer-generated sequence, and allocation concealment was maintained using a centralized online software. The study coordinators, surgeons, data collectors, outcome assessors, and patients were unaware of the randomization sequence at the time of recruitment. Once an eligible patient gave consent to join the study, the study coordinator input their information online and the allocation group was given. From this point onwards until the end of the study’s 30-day follow-up, only the study coordinator, the patient, and the Enterostomal Therapy nurse involved in the intervention were aware of the patient’s group allocation.

### Procedures

Patients randomized to the intervention group were scheduled to undergo 10 preoperative stimulation sessions during the 3-week period prior to ileostomy closure, similar to the Spanish protocol [13]. Each session was performed in the ambulatory clinic by a trained Enterostomal Therapy nurse. The lead investigator team disseminated an instructional video (https://www.youtube.com/watch?v=d8wl8RNoO48&t=139s) to participating sites to ensure a similar and safe procedure for each patient. During each session, the efferent limb of the ileostomy loop was canalized with an 18 Fr Foley catheter and infused with a solution comprising of 500 mL of normal saline mixed with 30 g of a thickening-agent (Nestle© Thicken-Up©). The Enterostomal Therapy nurses also recorded a daily stimulation diary including the amount of solution successfully irrigated, any complications or patient complaints that were observed, and the patient’s experience with evacuating the solution per anus. While the goal was for each patient to complete 10 sessions in the 3 weeks prior to surgery, this was a pragmatic trial; protocol violation was only considered (a priori) when < 7 sessions were completed, or > 2 weeks had elapsed from the time of the final bowel stimulation session to ileostomy closure (e.g., case cancellations with inability to reschedule imminently). Stimulation sessions that included < 500 mL of irrigated solution (e.g., due to significant reflux or abdominal cramps) were not considered protocol violations.

All other preoperative tests and interventions were similar between stimulation and control group patients. Patients were explained that they could cross-over to the control group arm (or withdraw from the study altogether) at any point from the intervention start date until their surgery, without exceptions.

Loop ileostomy closures were performed in a similar fashion at all sites. Surgery was performed under general anesthesia and preoperative intravenous antibiotics were given. A parastomal/elliptical incision was used for all cases, while a midline incision was performed when necessary to complete a difficult adhesiolysis or if intraoperative complications occurred. Anastomotic technique (stapled vs. hand-sewn) and skin closure (primary vs. secondary purse-string) was left to the discretion of the treating surgeon. Epidurals were not used, and patient-controlled analgesia was administered as per individual institutional protocols.

Postoperatively, all patients were managed within a site-specific ERP. While certain ERP elements differed between sites, all patients were given regular diet by postoperative day 1. Discharge criteria were similar at all hospitals: (1) tolerating oral diet; (2) no overt abdominal distension; (3) adequate pain control; (4) absence of fever or signs of deep surgical site infections; (5) ambulating at their baseline level; and (6) passage of flatus or stool.

### Outcomes

The primary outcome was postoperative ileus (POI), defined as an intolerance to oral food in the absence of clinical or radiological signs of obstruction, on or after postoperative day three, that either (a) required nasogastric tube insertion; or (b) was associated with two of the following: nausea/vomiting, abdominal distension, or the absence of flatus. Assessment of POI was performed by a trained, blinded assessor at each site. Patients were instructed not to reveal their group allocation to their surgeon, the postoperative team, or the POI assessor. Secondary outcomes included postoperative length of stay, time to first passage of flatus, overall 30-day morbidity, and 30-day readmission. In a subgroup of Quebec sites (three hospitals), bowel function, using the LARS (Low Anterior Resection Syndrome) Score, was also evaluated as an exploratory secondary outcome in patients whose index operation was a restorative proctectomy for rectal cancer.

### Sample size

In a previous observational cohort study including two of the participating sites, we observed a baseline incidence of POI after loop ileostomy closure of 16% [17]. Based on the Spanish trial [13], we anticipated a 3% incidence of POI among those who underwent preoperative bowel stimulation. With an alpha of 0.05 and 80% power, and accounting for a possible 5% loss in follow-up, 166 patients (83 in each arm) were required to detect a 13% absolute risk reduction with statistical significance. However, during the COVID-19 pandemic, recruitment was stopped for several months by local ethics boards due to concerns with patients coming to hospital 10 times preoperatively for research purposes. Once permitted to continue, recruitment had significantly slowed down, as ileostomy closure operations were deemed to be less of a priority at many sites, and were thus postponed or delayed indefinitely; some sites also transitioned to ambulatory ileostomy closure to minimize hospitalizations. As such, the lead investigators performed an unplanned interim analysis after 101 randomized patients, and the trial was terminated as superiority was demonstrated.

### Data analysis

Categorical variables (frequencies with percentages) were compared using *χ*^2^ or Fisher’s exact tests, and continuous variables (medians with inter-quartile ranges) using Wilcoxon rank-sum tests for non-parametric data. An unadjusted relative risk was calculated for the primary outcome, and a multiple logistic regression model was planned only if there was an imbalance between the two groups in baseline characteristics. A modified intention-to-treat analysis (excluding only those patients who were randomized but never underwent ileostomy closure) was performed for the primary analysis, and a per-protocol analysis was also performed to account for cross-overs. All statistical analyses were performed with R v3.5.1.

### Role of the funding sources

The Society of American Gastrointestinal and Endoscopic Surgeons and the Canadian Association of General Surgeons had no role in the study design, data acquisition and analysis, or manuscript preparation.

## Results

**Fig. 1:**
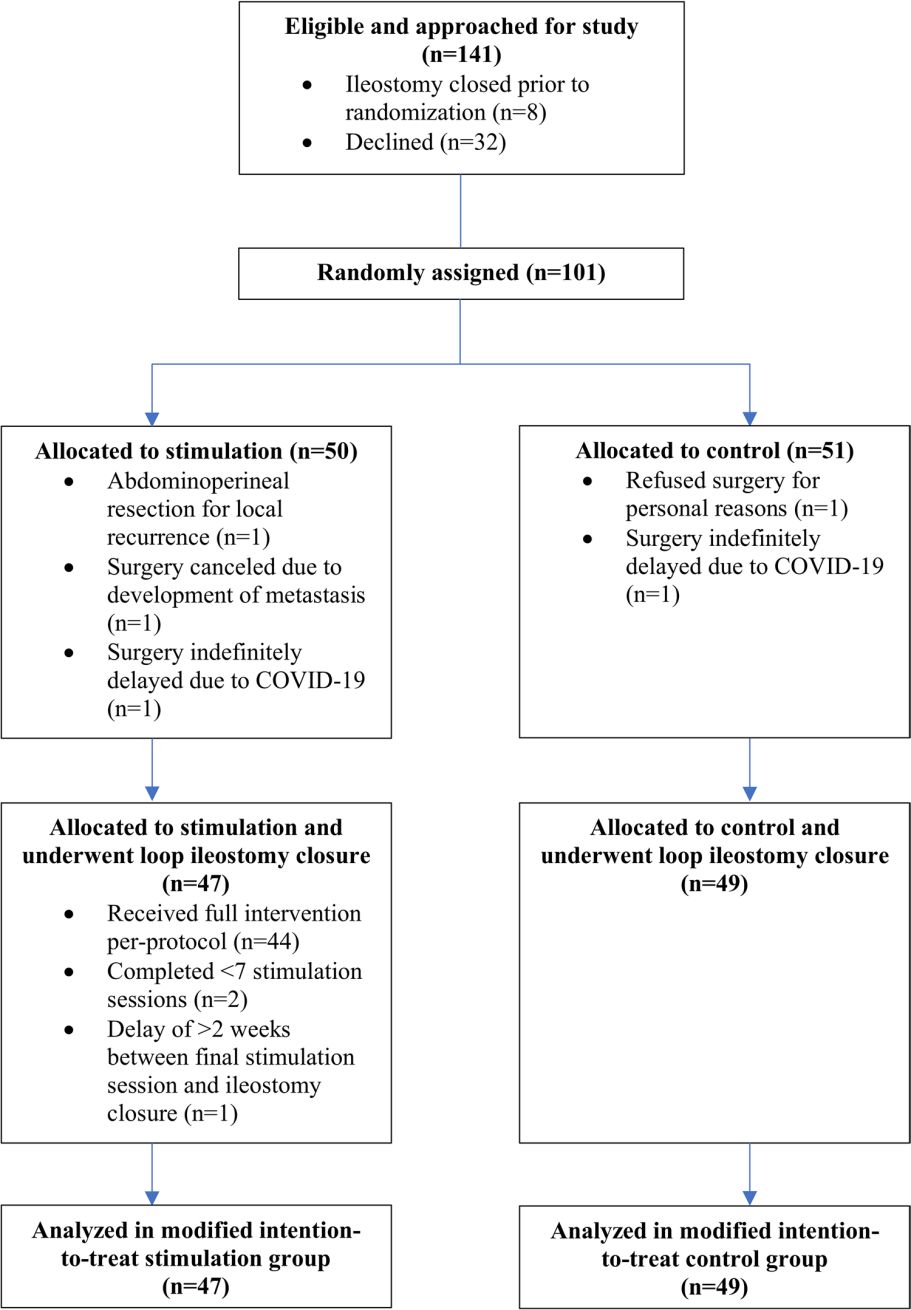
CONSORT flow diagram of randomized patients

Between January 2017 and November 2020, 141 eligible patients were identified and approached for inclusion into the study. Among them, 32 declined study participation and eight underwent ileostomy closure before they could be appropriately randomized. The most common reasons for decline were the time constraints and travel distance associated with the intervention. Of the 101 randomized patients, five never underwent ileostomy closure during the study period due to medical/personal reasons. Thus, 96 patients (47 stimulated vs. 49 control) were included and analyzed according to a modified intention-to-treat protocol (Fig. 1). All patients completed the 30-day follow-up.

Baseline demographics were well balanced between the two groups, including median age (stimulation: 62.0 years (55.5–71.0) vs. control: 64.0 years (53.0–71.0), *p* = 0.85), the proportion of male patients (57.4% vs. 53.1%, *p* = 0.82), and the proportion of patients with an American Society of Anesthesiologists score > 2 (31.9% vs. 22.4%, *p* = 0.41). Ileostomies were most commonly created as part of a colorectal resection for cancer (89.4% vs. 89.8%, *p* = 0.98), and the majority of index resections were performed with minimally invasive techniques (61.7% vs. 65.3%, *p* = 0.87). Nearly half of patients received adjuvant chemotherapy (46.8% vs. 38.8%, *p* = 0.56) and the median time between ileostomy creation and closure was similar between groups (6.7 months (5.0–11.0) vs. 7.3 months (5.4–10.1), *p* = 0.55) (Table 1).

**Table 1:** Baseline characteristics

Characteristic	Stimulation (*n* = 47)	Control (*n* = 49)	*p*
Demographics			
Age, years	62.0 (55.5–71.0)	64.0 (53.0–71.0)	0.85
Male	27 (57.4)	26 (53.1)	0.82
BMI, kg/m^2^	26.0 (23.7–29.2)	27.3 (23.2–31.0)	0.52
Cardiovascular disease	21 (44.7)	25 (51.0)	0.68
Pulmonary disease	5 (10.6)	3 (6.1)	0.67
Diabetes	5 (10.6)	8 (16.3)	0.61
Chronic kidney disease	2 (4.3)	6 (12.2)	0.30
Immunosuppressed	5 (10.6)	2 (4.1)	0.39
Smoking status	–	–	0.69
Current	5 (10.6)	5 (10.2)	–
Ex-smoker	14 (29.8)	11 (22.4)	–
Never	28 (59.6)	33 (67.3)	–
Charlson comorbidity score points	0.0 (0.0–2.0)	0.0 (0.0–2.0)	0.61
ASA Score	–	–	0.41
I/II	32 (68.1)	38 (77.6)	–
III/IV	15 (31.9)	11 (22.4)	–
Index operation			
Diagnosis	–	–	0.98
Malignancy	42 (89.4)	44 (89.8)	–
Benign disease	5 (10.6)	5 (10.2)	–
Neoadjuvant radiation	33 (70.2)	40 (81.6)	0.10
Surgical approach	–	–	0.87
Open	18 (38.3)	17 (34.7)	–
MIS	29 (61.7)	32 (65.3)	–
Timing of ileostomy	–	–	0.88
Before colorectal resection	3 (6.4)	3 (6.1)	–
During colorectal resection	41 (87.2)	43 (87.8)	–
After colorectal resection	3 (6.4)	3 (6.1)	–
30-day morbidity	29 (61.7)	23 (46.9)	0.21
Anastomotic leak	8 (17.0)	2 (4.1)	–
Postoperative ileus	10 (25.5)	10 (20.4)	–
Intersurgery period			
Adjuvant chemotherapy	22 (46.8)	19 (38.8)	0.56
Stomal morbidity	7 (14.9)	9 (18.4)	0.86
Hospital readmission	10 (21.3)	9 (18.4)	0.91
Intersurgery length, months	6.7 (5.0–11.0)	7.3 (5.4–10.1)	0.55

Data reported as median (Q1–Q3) or *n* (%), as appropriate

*BMI* body mass index, *ASA* American society of anesthesiologists, *MIS* Minimally-invasive surgery

Of 47 patients randomized to stimulation, 37 (78.7%) completed 10 preoperative stimulation sessions, 8 (17.0%) completed 7–10 sessions, and 2 (4.3%) completed < 7 sessions (1 patient crossed-over after 1 session due to abdominal cramps; 1 patient crossed-over before any session due to personal preference), resulting in a total of 410 stimulation sessions performed. Sessions lasted 25 min on average, and patients reported abdominal cramps in 27.6% of sessions. However, there were no major adverse events, and only four sessions (1.0%) were terminated early for poor patient tolerance. All patients experienced spontaneous rectal evacuations during the three-week period, with an average of 10 evacuations per patient after an average of 1.8 sessions (Table 2).

**Table 2:** Bowel stimulation intervention

Characteristic	
Patients randomized to stimulation	47
Completed stimulation sessions for each patient	–
10	37 (78.7)
7–9	8 (17.0)
< 7	2 (4.3)
Patients with > 2 week delay between final stimulation and closure	1 (2.1)
Total stimulation sessions, *n*	410
Duration of each session, minutes	25.3 (± 10.1)
Amount of solution injected per session, mL	437 (± 106.2)
Amount of overflow per session, mL	17.8 (± 12.2)
Major adverse events	0
Patient-reported cramps during session	113 (27.6)
Session terminated early due to poor patient tolerance (e.g., cramps/nausea)	4 (1.0)
Number of stimulation sessions to first evacuation (per patient) counted from, *n*	1.8 (± 2.2)
Number of evacuations (per patient) throughout the 3-week intervention period, n	10.0 (± 7.9)

Data reported as mean (± SD) or *n* (%), as appropriate

Median operative time was similar between the two groups (81.0 min (62.0–119.5) vs. 90.0 min (66.0–123.0), *p* = 0.46). The majority of patients had a stapled side-to-side functional end-to-end anastomosis (93.6% vs. 87.8%, *p* = 0.52) with secondary closure of the wound using a purse-string technique (76.6% vs. 69.4%, *p* = 0.64). Median narcotic use, measured in average daily postoperative morphine equivalents, was similar in both groups (7.5 mg (3.9–16.6) vs. 4.4 mg (2.6–8.3), *p* = 0.090) (Table 3).

**Table 3:** Ileostomy closure characteristics

Characteristic	Stimulation (*n* = 47)	Control (*n* = 49)	*p*
Operative time, min	81.0 (62.0–119.5)	90.0 (66.0–123.0)	0.46
Incision	–	–	0.57
Parastomal	43 (91.5)	46 (93.9)	–
Midline laparotomy-assisted	4 (8.5)	3 (6.1)	–
Small bowel anastomosis	–	–	0.52
Stapled	44 (93.6)	43 (87.8)	–
Hand-sewn	3 (6.4)	6 (12.2)	–
Skin closure	–	–	0.64
Primary (skin clips or sutured)	11 (23.4)	15 (30.6)	–
Secondary (purse-string)	36 (76.6)	34 (69.4)	–
Postoperative daily morphine equivalents, mg^a^	7.5 (3.9–16.6)	4.4 (2.6–8.3)	0.090

Data reported as median (Q1–Q3) or *n* (%), as appropriate

^a^In cases of postoperative ileus, narcotic use was only calculated on the postoperative days prior to meeting the outcome definition for ileus

In total, 15 patients developed POI (15.6%); two-thirds of POI cases required nasogastric tube insertion, while one-third met criteria for POI based on pre-defined symptoms. The incidence of POI was lower among patients randomized to stimulation (6.4% vs. 24.5%, *p* = 0.034; unadjusted RR: 0.26, 95% CI 0.078–0.87). Stimulated patients also had earlier median time to first flatus (2.0 days (1.0–2.0) vs. 2.0 days (2.0–3.0), *p* = 0.025), were more likely to pass flatus on postoperative day 1 (46.8% vs. 22.4%, *p* = 0.022), and had a shorter median postoperative hospital stay (3.0 days (2.0–3.5) vs. 4.0 days (2.0–6.0). The proportion of patients discharged by postoperative day 2 was also higher in stimulated patients (46.8% vs. 22.4%, *p* = 0.022). Of the three patients in the stimulation group who developed POI, one crossed-over to the control group and did not receive the full intervention (only one completed session). On per-protocol analysis, the superiority of bowel stimulation for POI reduction was even more pronounced (4.5% vs. 25.0%, *p* = 0.014; unadjusted RR: 0.18, 95% CI 0.043–0.72).

Overall 30-day morbidity was similar in both groups (12.8% vs. 30.6%, *p* = 0.062). There were very few non-POI morbidities, and only 2 reoperations (1 small bowel obstruction; 1 anastomotic leak). Thirty-day emergency room visits (6.4% vs. 6.1%, *p* = 0.97) and re-admissions (4.3% vs. 6.1%, *p* = 0.95) were also similar (Table 4). Among patients evaluated for postoperative bowel function, median LARS scores were similar between both groups (1-month LARS: 32.0 (11.8–38.3) vs. 34.0 (31.5–39.0), *p* = 0.34; 3-month LARS: 28.4 (15.8–32.0) vs. 31.0 (23.0–39.0), *p* = 0.26).

**Table 4:** 30-day morbidity following loop ileostomy closure

Characteristic	Stimulation (*n* = 47)	Control (*n* = 49)	*p*
Postoperative ileus	3 (6.4)	12 (24.5)	0.031
Nasogastric tube insertion	2 (4.3)	7 (14.3)	–
Return of flatus, days	2.0 (1.0–2.0)	2.0 (2.0–3.0)	0.025
Return of flatus POD #1	22 (46.8)	11 (22.4)	0.022
Overall 30-day morbidity	6 (12.8)	15 (30.6)	0.062
Superficial SSI	1 (2.1)	1 (2.0)	–
Organ space SSI	1 (2.1)	2 (4.1)	–
Anastomotic leak	0 (0.0)	1 (2.0)	–
Urinary tract infection	0 (0.0)	1 (2.0)	–
Acute kidney injury	0 (0.0)	2 (4.1)	–
Pneumonia	0 (0.0)	2 (4.1)	–
Myocardial infarction	0 (0.0)	0 (0.0)	–
Deep vein thrombosis	0 (0.0)	0 (0.0)	–
Pulmonary embolism	0 (0.0)	0 (0.0)	–
Clostridium difficile	0 (0.0)	2 (4.1)	–
Small bowel obstruction	1 (2.1)	0 (0.0)	–
Anastomotic bleeding	0 (0.0)	3 (6.1)	–
Reoperation	1 (2.1)	1 (2.0)	–
Postoperative length of stay, days	3.0 (2.0–3.5)	4.0 (2.0–6.0)	0.003
Discharged on/before POD #2	22 (46.8)	11 (22.4)	0.022
Emergency room visit	3 (6.4)	3 (6.1)	0.97
Readmission	2 (4.3)	3 (6.1)	0.95

Data reported as median (Q1–Q3) or *n* (%), as appropriate

*POD* postoperative day, *SSI* surgical site infection

## Discussion

The current study was the first multicenter randomized controlled trial to evaluate the impact of preoperative bowel stimulation via the efferent limb of the ileostomy on POI after elective loop ileostomy closure. Patients who underwent bowel stimulation had a significant reduction in the development of POI and a quicker return of postoperative bowel function, which culminated in a significant reduction in postoperative length of stay. Preoperative bowel stimulation was also demonstrated to be a safe intervention with no major adverse events and good patient tolerance.

The Spanish trial by Abrisqueta et al. was the only other randomized controlled trial to evaluate preoperative bowel stimulation before ileostomy closure [13]. The study design and procedures were very similar to the current study, including an almost identical definition for POI. The most notable difference between the two trials was the stimulation protocol. In the Spanish study, the ileostomy was irrigated with 500 mL of solution on consecutive days over a 2-week period; conversely, we allowed the intervention to take place over 3 weeks, and recorded details of each stimulation session in order to better describe our experience with this novel intervention. We felt that our pragmatic protocol, excluding only those patients with < 7 completed stimulation sessions and/or > 2 weeks elapsed before surgery, best reflects how the intervention could be implemented in clinical practice; it is less likely that someone would be able (or willing) to attend 10 sessions on consecutive days. Furthermore, there is no data on the optimal number of preoperative stimulation sessions, and fewer sessions may very well provide the same benefit. The Spanish trial reported a similar decrease in POI among stimulated patients (20% vs. 3%, *p* = 0.024), and mean postoperative length of stay was reduced by 2 days. Despite their positive findings, the trial was limited by its modest sample size and single-center recruitment, which prompted the undertaking of our multicenter trial.

The concept of functionally preparing the bowel before restoring intestinal continuity is appealing as there may be a biological basis to the effects of bowel stimulation. With a stoma, the defunctionalized segment of bowel undergoes a series of structural and functional changes that may contribute to the development of POI. Studies performed in animals have shown that defunctionalization of the ileum leads to atrophy of its villi and muscular layers [10]. This was confirmed in humans by Williams et al., who demonstrated a significant loss of muscular contractility and atrophy of intestinal villi following the creation of a diverting ileostomy [11]. Oh et al. also revealed lower concentrations of peptide YY secreted in the mucosa of the ileum and colon distal to a loop ileostomy, the function of which is to inhibit gastric motility and promote water and electrolyte absorption in the colon [12]. Thus, the absorptive capacity of the colon is reduced following reconstruction of the bowel. It is possible that stimulation via the efferent limb of the ileostomy may condition the bowel and reverse these structural and functional changes that occurred with diversion. Miedema and colleagues tried to explore this hypothesis in patients who underwent total proctocolectomy with diverting loop ileostomy, and demonstrated no effect on motor or absorptive capacity of the bowel with stimulation through the anus using a saline-based solution [18]. However, only six patients underwent transanal stimulation in their study, and given that all patients had an ileal-pouch anal anastomosis, there was very little bowel remaining to be stimulated. We hypothesized that bowel stimulation may also improve early postoperative bowel function as captured by the LARS score based on the same mechanism of action; however, in our small exploratory subset of patients, LARS scores at 1 and 3 months following ileostomy closure were similar in both groups.

As previously discussed, the rate of POI after ileostomy closure is surprisingly high, eclipsing 20% in several large, recently published series [4, 6]. Given the well-established clinical and financial sequelae of POI on both the patient and healthcare system [19], the 75% risk reduction associated with bowel stimulation renders it a very clinically-useful intervention. It is particularly appealing for those interested in ambulatory ileostomy closure, where POI can be an even more important source of morbidity when diagnosed late. In its current protocol, however, bowel stimulation can be rather cumbersome to complete. In addition to 10 preoperative hospital visits for the patient, it requires a trained healthcare professional to administer. Approximately 20% of patients who were approached for study inclusion refused to participate for logistical/personal reasons (e.g., lived too far from hospital, didn’t want to come so frequently, etc.). Additionally, there is a cost associated with the materials used and the hours allocated to the intervention. Unfortunately, quality of life data was not gathered in this study, and a formal cost-effectiveness analysis was not pursued. We would expect that the savings associated with reductions in POI and postoperative length of stay would outweigh the cost of the intervention, and this could be the subject of future studies. Furthermore, home-stimulation via community nurses and/or self-stimulation by engaged patients and their caregivers could be the next step in better realizing this intervention on a wider scale. For healthcare systems that do not have the resources to easily administer bowel stimulation, the intervention could also be limited to those at high risk of developing POI. Our group previously developed and validated a prediction model and web-based calculator for POI after loop ileostomy closure, which included five routinely available variables [17]. This calculator could help stratify patients and identify those that would benefit most from bowel stimulation.

This study has a number of strengths, including its randomized study design, the use of blinded outcome assessors, and its compliance with the CONSORT checklist, where applicable. However, there are several potential limitations. First, despite being the largest and first multicenter trial of its kind, the sample size was relatively modest, with only 96 patients analyzed. The trial was also terminated early largely due to the COVID-19 pandemic. However, an interim analysis demonstrated superiority for the intervention, and as such, risk for type II error was less relevant. Second, each participating hospital was allowed to use their own site-specific ERP which may have impacted postoperative outcomes. Studies have reported an association between the number of ERP components and risk for postoperative complications [20]. However, it would have been difficult to ask each site to adhere to a uniform ERP, and this was a pragmatic trial. Third, patients who declined study entry represent a possible source of selection bias, as non-participation may not have been at random. Finally, the intervention was limited to patients with residual colon in-situ, and these results are not applicable to patients with history of total proctocolectomy.

## Conclusions

In this international, multicenter, single-blinded randomized controlled trial, preoperative bowel stimulation via the efferent limb of the ileostomy reduced POI after elective loop ileostomy closure. Bowel stimulation is a promising intervention in this patient population, and may be incorporated into ERPs and prehabilitation programs going forward. Future studies should focus on better understanding the minimal number of stimulation sessions required to reduce POI and how the intervention may be delivered at home, in order to minimize hospital resources and maximize patient participation.

## Supplementary Information

Below is the link to the electronic supplementary material.

Supplementary file1 (DOCX 15 KB)
