# Supplementary material for: Bowel stimulation before loop ileostomy closure to reduce postoperative ileus: a multicenter, single-blinded, randomized controlled trial
Source: Surg Endosc. 2022 Aug 19;37(5):3934–43. doi: 10.1007/s00464-022-09510-5 (PMC9390101; doi:10.1007/s00464-022-09510-5)
Supplement: Supplementary file 1 — Supplementary file1 (DOCX 15 KB) [file 464_2022_9510_MOESM1_ESM.docx]

**Supplemental Table 1** Study patients by recruiting hospital

| **Hospital** | **Approached** | **Randomized** | **Underwent ileostomy closure** | **Stimulation** | **Control** |
| --- | --- | --- | --- | --- | --- |
| Jewish General Hospital, Montreal, QC, Canada | 64 | 53 | 51 | 23 | 28 |
| North Shore Hospital, Auckland, New Zealand | 24 | 17 | 15 | 5 | 10 |
| Montreal General Hospital, Montreal, QC, Canada | 16 | 11 | 11 | 8 | 3 |
| Rush University Medical Center, Chicago, IL, USA | 18 | 7 | 6 | 3 | 3 |
| North York General Hospital, Toronto, ON, Canada | 10 | 6 | 6 | 5 | 1 |
| University Health Network, Toronto, ON, Canada | 5 | 5 | 5 | 2 | 3 |
| St. Mary’s Hospital, Montreal, QC, Canada | 4 | 2 | 2 | 1 | 1 |
